# Why Try (Not) to Cry: Intra- and Inter-Personal Motives for Crying Regulation

**DOI:** 10.3389/fpsyg.2012.00597

**Published:** 2013-01-14

**Authors:** Gwenda Simons, Martin Bruder, Ilmo van der Löwe, Brian Parkinson

**Affiliations:** ^1^Department of Experimental Psychology, University of OxfordOxford, UK; ^2^Zukunftskolleg/Department of Psychology, University of KonstanzKonstanz, Germany

**Keywords:** crying, emotion regulation, inter-personal motives for regulation, intra-personal motives for regulation, emotion regulation strategies

## Abstract

This article discusses inter- and intra-personal motives for the regulation of crying, and presents illustrative findings from an online survey (*N* = 110) exploring why and how people regulate crying in their everyday lives. In line with current theorizing on emotion regulation and crying (e.g., Vingerhoets et al., 2000), we propose that emotional crying is regulated using both antecedent-focused techniques targeting the underlying emotion and response-focused techniques targeting the act of crying itself. Indeed, our survey respondents reported having used both antecedent- and response-focused strategies to either up-regulate or down-regulate their crying. Motives for crying regulation may be both inter- and intra-personal and may serve both immediate, pleasure motives, and future, utility motives (Tamir, 2009). Our findings suggest that down-regulation attempts are often driven by inter-personal motives (e.g., protecting the well-being of others; impression management) in addition to intra-personal motives such as maintaining subjective well-being, whereas up-regulation attempts are mostly driven by intra-personal motives. Further progress requires methodologies for manipulating or tracking regulation motives and strategies in real-time crying episodes.

Crying marks some of the most consequential and intensely emotional events in many people’s lives (Lombardo et al., 2001; Miceli and Castelfranchi, 2003) and may play a role in helping people to cope with such events (Vingerhoets and Scheirs, 2000). Besides its relevance for the individual, crying is also one of the most powerful inter-personal emotional signals showing that someone is “moved to an unusual depth” (Katz, 1999, p. 175). Although crying may “sometimes be used purposefully to manipulate people” (Vingerhoets and Scheirs, 2000, p. 144), it is usually seen as an authentic outburst of pure emotion. Despite being notoriously difficult to control, most people try to regulate their crying in at least some situations.

The causes of crying itself include situations involving rejection, personal inadequacy, pain and injury, separation, and criticism/rebuke (see Vingerhoets et al., 2001, for a brief overview) as well as certain positive events (e.g., birth of children, weddings). And although sadness, anger, anxiety, and frustration are the emotions most strongly associated with crying across a range of cultures (Vingerhoets et al., 2001), crying is also possible when people feel positive emotions such as relief, happiness, or joy (e.g., Miceli and Castelfranchi, 2003).

Some researchers argue that crying is not (necessarily) linked to emotional experiences. According to the behavioral ecology view, we might cry purely in order to communicate social motives to others, (e.g., because we perceive another person to be a likely source of comfort or help or because we want to draw attention to the injustice that has been done to us; Fridlund, 1991). Despite our focus in the present article on crying associated with emotions such as sadness and distress, we do not exclude the possibility of communicative crying. Indeed, it seems possible that communication is part of the function of the emotions associated with crying (e.g., Parkinson, 1996).

Although there has been some previous research into adult crying and its effects on health and well-being, little is yet known about the reasons for, and effects of, withholding tears or indeed encouraging them. Although numerous studies have investigated the regulation of (emotional) expressions and its effects in a great variety of contexts (e.g., Gross, 1998; Gross and John, 2003; Soto et al., 2011), there has been little systematic research of either direct regulation of crying or the regulation of emotions associated with crying (indirect crying regulation). Even less attention has been devoted to the motives underlying crying regulation.

In general, people regulate their experience and expression for a variety of reasons, such as increasing productivity at work, improving relations with others, and maintaining subjective well-being (Gross and Muñoz, 1995). Thus it appears that emotion regulation attempts serve to achieve both intra-personal and inter-personal effects (e.g., Evers et al., 2011; Parkinson and Simons, 2012). People do not always want to improve how they feel, and might sometimes be motivated to experience unpleasant emotions (e.g., for instrumental reasons, when those emotions promote the attainment of longer-term goals, Tamir, 2009). Correspondingly, crying and the emotions associated with it, might be down-regulated (inhibited) or up-regulated (increased) in line with either intra- or inter-personal motives.

This paper discusses potential strategies for the regulation of crying, perceived effects of crying, and the inter- and intra-personal motivations underlying crying regulation. Where relevant we will present initial evidence from an online survey which assessed crying and crying regulation in sad or upsetting situations in 31 male and 79 female respondents aged between 18 and 74 (*M* = 30.81, SD = 12.29). Most respondents resided in the UK (37%), the USA (31%), or another English-speaking country (9%). Sixteen percent of respondents resided in another European country and the rest (7%) lived elsewhere in the world.

The survey asked respondents to describe a situation in which they either felt the urge to cry or actually did cry. Respondents completed the survey in either the no-regulation of crying condition (*n* = 29); the up-regulation condition (*n* = 27), where they were asked about situations in which they encouraged crying; or the down-regulation of crying condition (*n* = 54), where they were asked to describe a situation in which they tried to inhibit their crying or prevent themselves from crying. All respondents indicated a medium to high urge to cry (*M* = 79.15, SD = 20.43 on a 100-point scale running from 0 = *not at all* to 100 = *extremely*). The extent to which respondents actually cried was lower (*M* = 48.88, SD = 37.96) and varied considerably across conditions. In both the no-regulation and up-regulation condition all respondents indicated crying during the event, whereas 12 of the down-regulation respondents managed to avoid crying altogether. The main reported causes of crying were: different types of loss [illness/death (19%); separation (20%); other loss (5%)]; conflict (26%); witnessing suffering (7%); movies; or music (7%), and the person’s psychological state (e.g., depression; 7%). Respondents also reported on their motivations for up-regulating, down-regulating, or not regulating their crying. Participants in the two crying regulation conditions (up-regulation and down-regulation) were additionally asked to describe how they regulated their crying and the emotions associated with crying (strategies). The event description and the description of methods of regulation were both open-ended questions (using an autobiographical narrative approach; e.g., Baumeister et al., 1990). Responses were coded by trained judges, and supplemented by quantitative self-report data (using rating scales and multiple choice questions) relating to regulation strategies and motivations for regulating – or not regulating – crying as well as questions about the social context in which crying occurred.

## How is Crying Regulated?

The regulation of crying associated with emotional experiences is perhaps best understood using the model of emotion regulation proposed by Gross and Muñoz (1995) which distinguishes two types of strategy: antecedent-focused regulation, in which the input to the emotional system is targeted (e.g., by situation selection) and response-focused regulation, in which the emotion program has been activated and the response tendencies which have been generated are modified by “strategies that intensify, diminish, prolong, or curtail on-going emotional experience, expression or physiological responding” (Gross, 1998, p. 225).

Consistent with Gross and colleagues’ approach, Vingerhoets and colleagues (Vingerhoets et al., 2000; Bekker and Vingerhoets, 2001) argue that crying can be regulated at both input and output stages of the emotional system. At the input stage, people might try to regulate their crying by regulating the emotions associated with crying. For example, an antecedent-focused strategy for crying regulation might entail avoiding situations that elicit the affective reactions that cause crying (situation selection), trying to change such situations (situation modification), shifting attention away from events that cause crying (attention deployment), or finding a different interpretation of these events (reappraisal). Similarly, people might use antecedent strategies to up-regulate crying, for example by focusing on the negative aspects of the situation or selecting a situation which they know will make them cry. For example, actors are commonly instructed to think of a sad memory in order to help them cry when required. By contrast, response-focused regulation strategies target crying directly either to down-regulate (*expressive suppression*, e.g., swallowing tears, trying to appear cheerful despite feeling sad, or trying to breathe normally) or to up-regulate (e.g., actors also sometimes make their tears flow by rubbing their eyelids with menthol or other irritants).

Bekker and Vingerhoets (2001) argue that person-related variables such as gender, personality, psychopathology, and socialization influence whether or not there is antecedent- or response-focused regulation of crying and which specific techniques are used. In addition, research has shown that reappraisal and other antecedent-focused regulation techniques are effective in decreasing emotional experience and expressive behavior without significant cognitive, physiological, or indeed inter-personal costs, whereas suppression and other response-focused techniques can lead to less satisfying social interactions (Gross et al., 2006). This suggests that antecedent-focused regulation serves inter-personal functions best.

Supporting the arguments from Vingerhoets and colleagues (Vingerhoets et al., 2000; Bekker and Vingerhoets, 2001), respondents in our survey reported having used both antecedent- and response-focused strategies to regulate their crying. Open-ended data from the survey revealed that they used strategies such as leaving or changing the situation (e.g., “I stepped into another room”), trying to reappraise the situation (e.g., “I focused on the positive aspects”), distracting themselves (e.g., “Tried to think of something else and concentrate on not being upset”), focusing on the situation’s negative or positive aspects (e.g., “I tried to increase crying by remembering happy times with her and thinking how I would never be with her again”), and actively suppressing (e.g., “I took deep breaths”; “I tried to get my face into shape”) or enhancing their crying (e.g., “I intensified my facial expression which made me feel even more sad and devastated”).

Quantitative survey data further confirmed that both types of strategy were used. For down-regulation, the mean ratings for response-focused strategies (*M* = 2.36, SD = 0.45) and antecedent-focused strategies (*M* = 2.38, SD = 0.82) were comparable (both rated on 5-point rating scales ranging from 1 = *not at all* to 5 = *a great deal*). For up-regulation, although the mean rating for response-focused strategies (*M* = 3.18, SD = 0.71) was significantly greater than for antecedent-focused strategies (*M* = 2.60, SD = 0.92), *t*(27) = 4.74, *p* < 0.001, both types of regulation strategies were reported by our respondents.

These preliminary findings need to be substantiated and extended in a more in-depth study of the strategies used to regulate crying. The survey relied entirely on retrospective self-reports about crying episodes. Although using such measures brings clear advantages when investigating crying and the strategies used to regulate crying (e.g., it allows the sampling of a wide range of personally involving, real life situations), there are also considerable disadvantages (Bylsma et al., 2011). Given the retrospective nature of these self-reports, people may, for example, report stereotyped memories, rather than giving an account of the actual events. In addition, given the sometimes very intense emotions experienced at the time of the crying event, memories might be distorted (e.g., Levine and Edelstein, 2009). The emotional nature of the original episodes might further cause respondents to either choose not to report certain events to protect themselves from remembering or even reliving the negative emotions associated with them. They may also alter their account to make it more socially desirable and as a result there might be unwillingness to report Machiavellian regulation (e.g., up-regulating of crying to get back at a partner). At the same time, there are problems in investigating crying regulation in more controlled settings, such as in the laboratory. First of all, eliciting crying in controlled settings raises important ethical concerns. Research must not expose participants to undue risk of harm (British Psychological Society, 2009; American Psychological Association, 2010); and exposure to potentially distressing events in order to elicit crying may indeed be harmful. Second, there are practical problems in devising induction techniques that elicit crying across a wide range of participants (Bylsma et al., 2011). However, notwithstanding these issues, it is recommended that future research supplements self-report data with more direct measures (for example real-time observation) in more controlled contexts. Diary methodology is also useful since event-contingent or daily reports on crying episodes are less likely to be affected by memory biases (e.g., Parkinson et al., 1995; Bylsma et al., 2011).

Drawing in part on Bekker and Vingerhoets’ (2001) adaptation of the regulation model (Gross and Muñoz, 1995), we propose that the extent to which crying is regulated and which strategy is selected to regulate it depend on the perceived effects of crying and regulation of crying, the salience of particular relational goals, regulation motives, and social norms concerning crying, the intensity of the underlying emotion, and person characteristics such as gender and personality.

## Intra-Personal and Inter-Personal Effects of Crying

To understand the inter- and intra-personal motivations for the deliberate regulation of crying and crying-related emotions, we first need to explore the functions of crying and especially what people believe the effects of crying to be. Our motivations to regulate or not regulate our crying are directly linked to our perceptions of the effects of crying on ourselves and the people around us, immediately and in the future. We regulate crying in order to achieve certain anticipated personal and inter-personal effects or to prevent or dampen effects that we anticipate would occur if we did not regulate. For example, research by Timmers et al. (1998) showed that women both cried more and anticipated more cathartic effects of crying than men. These authors also found that women were more likely to seek comfort when expressing sadness. This latter finding suggests that women may anticipate more positive inter-personal effects of crying than men do and consequently are less likely to inhibit their crying.

The effects of crying are also context-dependent. Crying occurs more frequently in some contexts, such as a funeral, compared to others, for example the office (e.g., Cornelius and Labott, 2001). People perceive there to be different consequences when crying whilst alone than with others and similarly, whether crying is up-regulated, down-regulated, or not regulated at all, depends in part on the presence or absence of others (e.g., Vingerhoets et al., 2001). Our survey showed that respondents were equally likely to be alone or with people that they knew when they up-regulated their crying or refrained from regulation. By contrast, down-regulation mainly occurred in the presence of person(s) known to respondents, but rarely when respondents reported being on their own. It follows that we should consider the effects that people perceive crying to have both for themselves and for those around them in order to understand the underlying motives for regulation.

### Intra-personal effects

Many people believe that it is good to cry, at least in certain circumstances and that holding back tears can have negative consequences for personal well-being (see Cornelius, 1986, for an informative review of articles in popular magazines). It is widely believed that crying can help people to recover from certain (emotional) events. “Sometimes it’s better to get it all out,” as one of our respondents put it. Indeed, it is widely assumed that crying can be healthy and restorative (e.g., Efran and Spangler, 1979; Kraemer and Hastrup, 1988). A similar intra-personal function of crying is to alleviate depression. Relatedly, crying is often observed during psychotherapy, and is generally seen by therapists as a potentially cathartic discharge of affect (e.g., Cornelius, 2001; Nelson, 2008).

The reported benefits of crying for affect, when they occur, appear to depend on the mood characteristics of the person crying (dispositional positive and negative mood) and the social context in which crying occurs. In their diary study involving 97 women, Bylsma et al. (2011) found that those women who were high on dispositional negative mood (i.e., average negative mood across the whole diary period) and low on dispositional positive mood, reported more crying episodes, and a higher urge to cry. They further found that in their study one person being present during the crying episode improved the mood of the crier afterward whereas the presence of multiple others had a negative impact. In other words, there is evidence that the intra-personal effects of crying regulation, like its inter-personal effects, are variable rather than fixed.

If crying releases or purges negative affect, then its inhibition may correspondingly worsen mood, well-being, and (mental) health (in contrast to potential positive effects of up-regulation). Indeed, there is evidence that the routine down-regulation of emotions (and of crying in particular) undertaken by health professionals and police officers may ultimately lead to burnout (e.g., Bakker and Heuven, 2006). However, it is important to distinguish between the immediate effects of the inhibition of a single crying episode and the long-term effects of the chronic inhibition of crying (Vingerhoets and Bylsma, 2007), and it might be the latter in particular, which has negative effects for well-being (Bakker and Heuven, 2006). It is also possible that these effects depend on the individual, organizational, and environmental factors that lead workers to engage in regular down-regulation in the first place rather than down-regulation itself.

Crying is further thought to be associated with the reduction of accumulated tension and physiological arousal (e.g., Efran and Spangler, 1979; see Vingerhoets et al., 2000, for an overview). Thus, inhibition of crying may lead to increased autonomic activation (Gross, 1998), bringing adverse consequences for physical health in the longer-term (Vingerhoets et al., 2000). These adverse consequences may depend on the strategies deployed to regulate crying. Prior research suggests that response-focused regulation of emotions in particular is accompanied by increased sympathetic nervous system arousal, due to regulatory effort as well as disruption of the usual tension-reduction process (Gross, 1998). It follows that when a response-focused technique is used to inhibit crying, there should be a marked increase in sympathetic nervous system arousal. Response-focused regulation can also bring cognitive costs such as interference with processing of emotional stimuli (e.g., Richards and Gross, 2000). To the extent that these cognitive consequences reduce the emotional power of perceived events, suppression of crying may bring beneficial as well as detrimental consequences for affect.

Finally, crying can influence how criers perceive themselves. Crying is often associated with being weak or incompetent and people might sometimes down-regulate their crying to be able to see themselves as competent. On the other hand, in certain circumstances people associate crying with being a warm person, who is not afraid of showing their emotions. Thus people may refrain from regulating crying or even up-regulate in order to achieve or maintain a warm self-image. Relatedly, as we will see in the next section, people might want others to perceive them as a warm or competent person and regulate their crying to manage the image that others have of them since this in turn may affect how they feel about themselves.

Although the above review is by no means exhaustive, the documented intra-personal effects of crying clearly suggest that the regulation of crying can have effects on mood, as well as direct and long-term effects on physical and mental health (Gross, 1998; Vingerhoets et al., 2000). It further can affect people’s image of themselves. However, the regulation of crying is not only driven by intra-personal motives relating to improvement in well-being and mood or future outcomes related to self-concept concerns (see also Tamir, 2009). People may regulate their crying in order to achieve certain inter-personal effects, even if they believe that the regulation of crying may have negative intra-personal consequences.

### Inter-personal effects

As discussed above, people may regulate crying because of their beliefs about and experiences of the consequences of crying and not crying. Some of the anticipated consequences of crying that motivate regulatory efforts are effects that mainly operate on other people rather than on the self. However, it is also worth noting that one of the reasons for caring about inter-personal effects is that other people’s reactions have effects on the crying person too. As discussed in the next section, people may regulate crying because they anticipate immediate rewards or less direct instrumental benefits (Tamir, 2009) and some of these rewards and instrumental benefits may be provided by other people’s reactions to crying. Thus, inter-personal effects may mediate intra-personal effects as well as vice versa, so that the two may become inextricably interlinked.

Our main focus in the present section is on inter-personal effects that depend on the perceived emotional meaning of crying. Perceivers tend to see crying as an outpouring of authentic emotion which may or may not be appropriate in a particular situation. Many inter-personal effects of crying depend on other people’s interpretations of its emotional implications. In particular, people may believe that others will suffer as a consequence of seeing that they are upset enough to cry. For example, one respondent in our survey indicated that she feared that her crying would cause those around her to become sad or upset as well (“if the others see me crying it will possibly make them feel even worse”) or that their tears might induce contagious crying (“if my daughter saw me crying, she would start as well”). Hendriks et al. (2008) found that participants experienced more negative emotions (but offered increased support) when imagining a crying person than a non-crying person. Recent research has also shown that one person’s sadness displays can lead to convergent responses in another person (Bruder et al., 2012).

One model that might help explain why crying can lead to different possible behavioral and affective responses from others was proposed by Goubert et al. (2005) to account for people’s affective and behavioral responses to observing pain in other people. According to these authors, the empathic sense of another’s pain and associated affective responses depends on features of the incoming stimulus (including the observed person’s facial or verbal expressions and cues from the environment), and features of the observer, such as the observer’s learning experiences and shared knowledge. The observer’s affective responses may be either oriented toward the observers themselves (e.g., distress or anxiety) or oriented toward the observed person (e.g., sympathy with the person in pain), and these affective responses will in turn affect the observer’s behavioral responses. Similarly we might expect corresponding factors to influence the empathic sense of another’s distress or sadness at the sight of someone crying and as a result the person observing the crier might become distressed themselves or might experience sympathy with the crier.

Crying can influence other people’s impressions of the crier’s personal characteristics as well as their emotions. For example, criers may be perceived by others as weak, sensitive, or powerless. Consequently, people might anticipate being seen as more competent (e.g., *capable*, *confident*; Cuddy et al., 2008) by others when down-regulating or avoiding crying. Indeed, one explanation for our respondents reporting being relatively more likely to down-regulate rather than up-regulate crying when with others is that they believed that their crying would negatively affect other people’s impressions of them. People might worry about these social reactions that in turn can make them feel bad. Correspondingly, they may feel ashamed about crying in front of others, or expect to be taken less seriously if they do cry. This provides another example of how intra-personal effects may depend on real or anticipated inter-personal ones. On the other hand, people might anticipate being seen as a warmer, more emotional person when they do cry in particular circumstances (e.g., when witnessing suffering) and might allow crying or even up-regulate their crying as a result.

Several of our respondents indicated that they tried not to cry in a class/work situation because they felt it might give others a negative view of them. Indeed, research suggests that crying can come with unwanted inter-personal consequences in many social contexts (Hendriks and Vingerhoets, 2002). According to Efran and Spangler (1979), although crying is considered a healthy behavior there are social taboos related to the crying of specific people in specific situations. There appears to be a stigma attached to crying, particularly for people who are in charge of others or who occupy positions of responsibility (Efran and Spangler, 1979). For example, Wagner et al.’s (1997) found that medical students reported being ridiculed or shouted at when they cried during their hospital shift.

More generally, negative social consequences may result from failing to abide by so-called display rules, which specify when and where it is appropriate to express certain emotions (e.g., Fischer et al., 2004). The nature of these display rules depends on the cultural environment (Matsumoto et al., 2008) and on the position a specific person occupies in it (Becht et al., 2001). For example, Van Hemert et al. (2011) argue that crying, like other forms of expressiveness, is influenced by cultural norms prescribing how, when, and where it is appropriate to express the associated emotion. In their research, they found that individuals living in countries that allow more freedom of expression of individual feelings (i.e., democratic and individualistic countries) cry more often than individuals in more restrictive countries. However, some cultures also actively encourage crying by certain people in certain public situations. For example at Iranian funerals it is very much expected that the mourners, especially women, weep, and wail at the home of the deceased, at the funeral, and during various services at the mosque (Chosky, 2006).

These display rules depend on social roles as well as culture. For example, from an early age, boys are often told that they should not cry across a wide variety of situations (Big boys don’t cry!; e.g., Camras, 1986; Simons and Bruder, 2012). In addition to familial socialization, gender differences in the expression of sadness and distress through crying may arise from differential peer socialization histories where the expression of sadness and pain is encouraged among girls through supportive inter-personal reactions from their peers but in boys is met with discouraging peer responses (e.g., Zeman and Shipman, 1996). Thus, showing tears may lead to more negative social consequences among men than women. More generally, the appropriateness of crying depends on a range of personal attributes (including gender) and their relation to the surrounding context, including one’s particular relationship with the other person(s) present.

It is important to remember here that crying’s inter-personal effects do not only depend on its emotion-expressive aspects. As mentioned above, crying may serve a number of inter-personal or social functions, including communicating vulnerability and appealing for help (e.g., Fridlund, 1994; Parkinson, 2005), that do not necessarily depend on others’ perceptions of underlying emotions. For example, crying can be seen as a form of attachment behavior designed to elicit care-giving responses from important others (e.g., Nelson, 2008). Thus, people who down-regulate crying may receive less social support than those whose crying remains unregulated or those who up-regulate their crying. Indeed, participants in a vignette study by Hendriks et al. (2008) reported that they would give more emotional support and express less negative affect to a crying person compared to a non-crying person.

As the above discussion shows, the inter-personal effects of crying are varied. Crying might cause other people to experience distress. It might also affect how other people view us, elicit certain social reactions (e.g., being pitied), or induce disapproval because it conflicts with display rules. Alternatively, crying might communicate our need for help. Given the wide range of actual and perceived effects of crying and crying regulation discussed above, it seems likely that the motivations behind crying regulation will be correspondingly diverse.

## Motives for Crying Regulation

The previous section showed that crying may have immediate and cumulative inter- and intra-personal effects. We now turn to the motives behind crying regulation, many of which may be understood by reference to anticipation of these effects. In other words, crying regulation may serve the function of achieving immediate or future intra- and inter-personal effects.

Tamir’s (2009) instrumental theory of intra-personal emotion regulation provides a useful framework that may be extended to the understanding of the motives behind crying regulation. Her basic distinction is between pleasure and utility motives. The pleasure motive concerns the immediate situation and aims to achieve more positive affective states, whereas the utility motive focuses on future outcomes and promotes emotions which further the individual’s goals but are not necessarily immediately pleasurable. Based on this distinction, Table 1 gives examples of potential intra- and inter-personal motives for both the down- and up-regulation of crying classified according to whether the focus is on either the immediate situation or the (near) future. We discuss the motives fitting the resulting eight cells of the table in the following sections.

**Table 1:** **Examples of motives for intra- and inter-personal regulation of crying classified according to their dependence on the pleasure or utility motive**.

	Down-regulation		Up-regulation/no-regulation	
	Focus on immediate situation/pleasure	Focus on future outcomes/utility	Focus on immediate situation/pleasure	Focus on future outcomes/utility
Intra-personal	(a) Avoid or diminish experience of negative emotion	(b) Self-concept concern: see oneself as a competent person	(e) Vent feelings, achieve catharsis	(f) Self-concept concern: see oneself as a warm person
Inter-personal	(c) Avoid attention, avoid social reactions that make one feel bad (e.g., pity or ridicule)	(d) Reputational concerns/elicit appraisals of competence	(g) Attract attention, elicit positive social reactions (e.g., help provision)	(h) Reputational concerns/elicit appraisals of warmth

### Intra-personal motives for down-regulation

People may inhibit crying in an attempt to avoid or diminish the experience of negative emotions. Our survey respondents frequently endorsed intra-personal motives focusing on the immediate situation (see Table 1, cell a) for the down-regulation of crying (e.g., “I did not want to increase the negative feelings I was experiencing”; 57% of respondents, see also Table 2). An intra-personal motive for crying down-regulation which focuses more on future outcomes is the wish to see oneself as a competent person (Table 1, cell b). For example, in our survey 41% of respondents endorsed the statement (Table 2) that they down-regulated because “I felt that I would think of myself as weak.”

**Table 2:** **Frequencies of motivations endorsed in the survey**.

Why did you down-regulate your crying? (*N* = 54)		(%)	
Intra-personal	Because I did not want others to know how I felt	59	
	Because I did not want to increase the negative feelings I was experiencing	57	
	Because I felt it was inappropriate for me to cry	50	
	Because I felt that the experience of crying would increase my distress	44	
	Because I did not want to cause additional distress to myself	44	
	Because I felt that I would think of myself as weak	41	
	Because I felt that I would think of myself as overly emotional	28	
Inter-personal	Because I felt that others’ reactions would increase my distress	54	
	Because I did not want to cause distress to others	48	
	Because I felt that others would think of me as overly emotional	42	
	Because I felt that others present would consider it inappropriate for me to cry	39	
	Because I felt that others would think of me as weak	37	
	Because I did not want to increase negative feelings others were experiencing	33	

**Why did you up-regulate (*N* = 29)/did not regulate your crying (*N* = 27)?**		**Up (%)**	**No (%)**

Intra-personal	Because my feelings were so strong that I could not avoid shedding tears/tearing up	72	96
	Because I felt it was appropriate for me to cry	66	52
	Because I felt that the experience of crying might decrease my distress	62	56
	Because I felt that I needed a good cry	59	41
	Because I wanted to increase the negative feelings I was experiencing	24	4
	Because my attempts to prevent myself from shedding tears failed	14	41
	Because I felt that I would think of myself as non-emotional if I did not	6	7
Inter-personal	Because I wanted others to know how I felt	34	22
	Because I needed support from other people	24	26
	Because I felt that others would think of me as non-emotional if I did not	21	4
	Because I felt that others present would consider it appropriate for me to cry	17	11
	Because I wanted to increase negative feelings others were experiencing	14	4
	Because I felt that others’ reactions would decrease my distress	10	15

### Inter-personal motives for down-regulation

Despite the possible intra-personal motives discussed above, our survey suggests that down-regulation of crying occurs mainly when people are in the presence of others, thus implying that anticipated inter-personal effects of crying may be more relevant to down-regulation motives. For example, people might inhibit their crying to avoid attention and social reactions that increase bad feelings such as being ridiculed or pitied (Table 1, cell c). For understandable reasons, medical students in Wagner et al.’s (1997) study who reported more negative social consequences of crying also reported less actual crying, probably because of their attempts to inhibit it. In our own research, more than half (54%) of our respondents indicated that they down-regulated “Because I felt that others’ reactions would increase my distress” (see also Table 2).

Another set of inter-personal motives relates to concerns about how crying might affect other people’s perceptions of the person crying and focuses on future outcomes (self-presentation/reputational concerns; Table 1, cell d). In our survey, 42% of respondents reported that they down-regulated crying “Because I felt that others would think of me as overly emotional” (see also Table 2). One respondent gave the following account of crying down-regulation in the workplace:

I was at work and received news that my grandmother had been diagnosed with cancer. I was due to meet clients immediately after, so tried to focus on the task at hand as I felt that if I was to start crying it would be difficult to stop. I absorbed myself in my work as a means of distraction and then cried when I got home and saw my family.

She further states: “I did not want clients to see me cry as it would interfere with work and may seem unprofessional.”

### Intra-personal motives for up-regulating or not regulating crying

Crying up-regulation or unregulated crying seems to occur mainly when the focus is on achieving catharsis in the immediate situation (Table 1, cell e). Those reporting up-regulation of crying or absence of regulation in the survey (see also Table 2) chiefly endorsed intra-personal motives (e.g., “I felt that I needed a good cry”; 59 and 41% of respondents, respectively) or referred to their inability to stop crying (e.g., “Because my feelings were so strong that I could not avoid shedding tears/tearing up”; 72 and 96% respectively), although unbridled crying or up-regulating of crying may also be motivated by future outcomes such as wanting to see ourselves as a warm or emotional person (Table 1, cell f). For example, a small proportion of respondents (6% in the up-regulation and 7% in the unregulated crying condition) endorsed the statement “Because I felt that I would think of myself as non-emotional if I did not” (see also Table 2).

### Inter-personal motives for up-regulating or not regulating crying

However, unregulated or up-regulated crying may also occur for inter-personal reasons, both when the focus is on the immediate situation (e.g., “Because I wanted others to know how I felt”; endorsed by 22 and 34% of respondents respectively, see also Table 2) and when the focus is on the future e.g., “Because I felt that others present would consider it appropriate for me to cry” endorsed by 11 and 17% of respondents respectively, see also Table 2). For example, one respondent described how he urged himself to cry in order to show his girlfriend how upset she made him (inter-personal motive focused on the immediate situation; Table 1, cell g). Another respondent described how he could not cry during the funeral of his mother-in-law and how he actively tried to think of it as his own mother being dead so he would have the appropriate emotions when doing a reading at the funeral (Reputational concerns, Table 1, cell h).

### Other inter-personal motives for crying regulation

An inter-personal motive which follows from the inter-personal effects discussed in the previous sections is to modify the effects of our emotional displays on others. Concern for others’ well-being does not fit neatly into the categories of motives listed in Table 1, as the focus is not so much on achieving positive affective state or specific future outcomes for oneself. However, survey respondents endorsed motives to reduce or change the effects of crying on what other people (might) experience (e.g., “I did not want to cause distress to others”; 48%). It appears that, in the case of crying at least, Tamir’s (2009) classification can be extended to include motives to attain reward and instrumentality for other people (although these too may indirectly be intra-personally motivated). One of our male respondents indicated that upon the death of the husband of a cousin “I did not allow myself to cry because it would have been no help for them. They needed some stability, solace and help – not even more tears.”

Another respondent also talks about the effect his crying would have on other people and how in the circumstances it was not appropriate for him to cry:

Usually I have healthy barriers between myself and people who come to me with their difficulties (it is part of my job) and am aware enough of my own trigger points to not be affected by others’ emotions, but about a month ago a man (section redacted to retain participant confidentiality) was talking to me about his daughter and started to cry and I found myself welling up with him. It is not appropriate for me to sit there weeping with the people I support so I had to suppress the tears and get myself back to a neutral place to be better able to support him.

This latter example appears to include concerns both for the other person and the respondent himself (reputational concerns).

### Multiple motives possible

Although we have given frequencies of respondents from our survey endorsing particular motives for each of the cells, this should not be interpreted as evidence that people always have only a single motive for regulating their crying. In fact, someone might be motivated to down-regulate their crying for both inter- and intra-personal motives focused on the immediate situation as well as the future and thus endorse a number of different motives (including: “Because I did not want to cause distress to others” and “Because I did not want to increase the negative feelings I was experiencing” – a combination seen in 33% of down-regulation cases). Similarly, certain motives belong in more than one of the different cells. For example, down-regulation motivated by the desire to avoid ridicule serves both to make ourselves feel better and to improve our image in the eyes of onlookers.

## Conclusion

In the present article we have discussed the motives for crying regulation, the (perceived) effects of crying and crying regulation, and the potential strategies used for the regulation of crying. We have presented some initial findings suggesting that crying is indeed regulated both by antecedent- and response-focused techniques as suggested by Vingerhoets and colleagues (Vingerhoets et al., 2000; Bekker and Vingerhoets, 2001). Future research should establish more conclusively what kinds of strategies are used in crying regulation and which factors influence the choice of strategy. We propose that the extent to which crying is regulated and which strategy is selected depend on the presence or absence of specific individuals, the salience of particular relational goals, regulation motives, cultural, and social norms concerning crying, the intensity of the underlying emotion, and person characteristics such as gender and personality (see Bekker and Vingerhoets, 2001 for a discussion of some of these factors). Given the potential negative consequences of response-regulation for inter-personal interactions (e.g., Gross et al., 2006), we predict that inter-personal motives for crying regulation will be more strongly associated with antecedent-focused strategies (e.g., reappraisal), and that intra-personal motives will be more strongly associated with response-focused strategies such as suppression. We also assume that these relationships will be moderated by the nature of the activated social goals for regulating crying. Unfortunately, the data from our survey do not permit direct exploration of these hypotheses because many participants reported both inter-and intra-personal motives, or stated that they had used both antecedent- and response-focused regulation strategies. Either a survey using a larger sample or an experimental study would be needed to examine these hypotheses.

Further, more research is needed to gain insight into the underlying motivations for crying regulation, including the expected effects of crying on factors such as own and others’ well-being, self-concept, and self-presentation. The effects of crying and crying regulation discussed in this article indicate that crying regulation might occur to modify both inter- and intra-personal consequences and that we can distinguish between pleasure and utility motives (immediate and future effects; Tamir, 2009). On an inter-personal level, people are not only concerned with how crying affects how they are seen by others around them but also how their crying affects other people, in terms of how it makes these other people feel. Further, whereas down-regulation of crying appears to result from both inter- and intra-personal motives, the up-regulation of crying is very much done for the benefit of the crier, although there are exceptions, for example when people think that crying is expected (e.g., at funerals). Importantly, it may depend on the situation and the social norms governing the situation whether, for example, reputational concerns lead one to down- or up-regulate crying.

Although this article has mainly addressed crying from an emotion expression view, the behavior ecology view (Fridlund, 1991) should also be considered when interpreting present and future findings. According to this account, social motivational variables should determine whether there is an impulse to cry in the first place. Thus, rather than there being an “impulse” to cry which is subsequently modified by regulation, such an account would hold that inter-personal concerns enter the picture more directly. Since our survey asked participants about situations where they felt the urge to cry or actually cried, the instructions pre-supposed an emotion expression view of crying and our data therefore cannot be used to fairly distinguish between these two accounts in the context of crying behavior. However, our survey data did include respondents who reported up-regulating or not regulating their crying in order to communicate their pain to others, to manipulate the situation to their advantage, or – in the case of one respondent who was angry with his partner for not taking care of him – to use crying as a sort of revenge: “as soon as I get angry with her, she often starts crying, and in this situation, I encouraged myself to cry.”

The evidence we have presented in this paper is based on people’s retrospective self-reports, which, as we have discussed, may be distorted by memory, self-protective motives, and self-presentational biases. Additional research using a wider range of methods is needed to gain insight into underlying motivations for crying regulation, including the expected effects of crying on factors such as own and others’ well-being, self-concept, and self-presentation. It is also important to clarify how these motivations vary across situations and persons. Future studies should combine the use of self-reports with other methodologies such as manipulating or tracking of regulation motives and strategies in real-time crying episodes in reaction to specific stimuli (e.g., crying-inducing films or vignettes) in more controlled settings. In this way, the effect of certain variations in social context (such as who is present) can be controlled. However, as discussed above, the use of controlled settings such as laboratories also comes with disadvantages, not least ethical considerations that restrict the possibilities to induce crying and other problems related to their ecological validity (e.g., awareness of being watched or video-taped while crying). Further, in order to obtain records of crying and crying regulation in daily life, the use of diaries to record crying and crying regulation episodes as they happen should help to avoid retrospective biases and memory errors (e.g., Parkinson et al., 1995; Bylsma et al., 2011).

The regulation of crying and its underlying motives is relatively unchartered terrain, which, given the perceived and actual substantial effects of crying, needs to be urgently explored. We believe that the initial empirical evidence and the theoretical outline presented here can help guide the next steps in this exciting research area.

## Conflict of Interest Statement

The authors declare that the research was conducted in the absence of any commercial or financial relationships that could be construed as a potential conflict of interest.
